# Immune factors have a complex causal regulation on pulmonary fibrosis: Insights from a two-sample Mendelian randomization analysis

**DOI:** 10.1097/MD.0000000000036781

**Published:** 2023-12-29

**Authors:** Zhiyu Tian, Zhanliang Jiang, Shaodan Hu, Li Shi

**Affiliations:** a Changchun University of Traditional Chinese Medicine, Changchun, Jilin Province, China; b The Affiliated Hospital of Changchun University of Traditional Chinese Medicine, Changchun, Jilin Province, China.

**Keywords:** immune cells, Mendelian randomization, observational study, pulmonary fibrosis

## Abstract

Pulmonary fibrosis is a chronic, progressive lung disease characterized by excessive scarring of lung tissue, and its pathophysiological mechanisms have not been fully elucidated. Immune cells play a key role in many diseases, and this study aims to explore the causal link between immune cell characteristics and pulmonary fibrosis using Mendelian randomization. Utilizing the public GWAS database Open GWAS, this study collected whole-genome association study datasets of peripheral blood immune phenotypes and summary data of GWAS related to pulmonary fibrosis. Through Mendelian randomization (MR) analysis, we identified single nucleotide polymorphisms (SNPs) significantly associated with immune traits as instrumental variables. After pleiotropy and heterogeneity tests, causal effects were assessed using methods such as inverse-variance weighted (IVW), weighted median, and MR-Egger. Comprehensive MR analysis indicated a significant causal relationship between various immune cell types, including regulatory T cells (Tregs), natural killer (NK) cells, and specific monocyte subgroups, with the risk of pulmonary fibrosis. Specifically, phenotypes such as Activated & resting Treg %CD4+, CCR2-positive monocytes, and CD16-CD56 positive NK cells were associated with a reduced risk of pulmonary fibrosis. In contrast, CD8 + T cell subgroups were associated with an increased risk. This study provides evidence of a causal relationship between immune cell characteristics and pulmonary fibrosis, highlighting the protective role of regulatory T cells and specific NK cell subgroups, as well as the potential harm of CD8 + T cell subgroups. These findings offer new insights into the immunoregulatory mechanisms of pulmonary fibrosis and the development of novel therapeutic strategies.

## 1. Introduction

Pulmonary fibrosis is a severe lung condition characterized by excessive fibrosis of lung tissues, leading to pulmonary function decline and potential respiratory failure.^[1]^ This condition often affects middle-aged and older individuals, manifesting as persistent dry cough and progressively worsening breathlessness. Although pulmonary fibrosis can be caused by various factors, the exact cause of idiopathic pulmonary fibrosis (IPF) often remains unknown. Treatment primarily focuses on symptom relief and slowing disease progression rather than cure.^[2]^ Advances in modern medicine have deepened our understanding of the disease’s molecular mechanisms, but the complexity of pulmonary fibrosis means its pathogenesis is still not fully clear, limiting the development of effective treatments.

Studies have shown that abnormalities in the immune system, including macrophage activation and aberrant microRNA secretion, are key to the development of pulmonary fibrosis. This offers a new direction for treatment, but due to the disease’s complexity and individual variability, treatment must be personalized, taking into account the patient’s genetic background and disease characteristics.^[3]^ Pulmonary fibrosis is a pathological process present in various clinical conditions, constituting a spectrum of diseases ranging from connective tissue disease-associated fibrosis to the more intractable idiopathic pulmonary fibrosis. IPF is a progressive fatal condition, typically with limited response to immune-targeted treatments.^[4]^ While animal model studies have provided potential therapeutic targets, translating these findings to clinical application requires a deeper understanding of disease heterogeneity and common fibrotic pathways. The diagnosis and treatment of pulmonary fibrosis are complex, partly due to its pathological diversity; nonspecific pulmonary fibrosis can radiologically and histologically mimic IPF, demanding precise discrimination by clinicians.^[5]^ Additionally, while various genetic variants have been identified as related to the risk of pulmonary fibrosis, understanding the role of these genetic and epigenetic factors is still evolving, further complicating treatment. Broadly, pulmonary fibrosis is viewed as a dysregulated repair process, where fibrosis can persist even when traditional inflammatory pathways are suppressed. The recruitment, activation, apoptosis, and clearance of key effector cells are highly conserved and tightly regulated following tissue injury.^[6]^ In the study of the immune system, macrophage polarization and the microRNAs they produce play a crucial role in regulating inflammation and immune responses, with M1/M2 macrophage phenotypes corresponding to pro-inflammatory and pro-fibrotic properties. MicroRNAs released by M2 macrophages may promote pulmonary fibrosis, making these non-coding RNAs potential therapeutic targets.^[7]^ Considering these findings, pulmonary fibrosis is a complex pathological process involving multiple levels and factors, requiring an in-depth understanding of its immune mechanisms for treatment. Future research should focus on how knowledge of immune responses can be used to develop new treatment regimes, especially for those patients with IPF who are insensitive to conventional treatments.

In modern epidemiological research, observed associations are often influenced by confounding factors, reverse causality, and various biases, making it extremely complicated to infer causal relationships from statistical correlations. Traditional randomized controlled trials (RCTs) are considered the gold standard for causal inference but are not always feasible due to cost, ethical restrictions, and practical difficulties. Against this backdrop, Mendelian Randomization (MR) has emerged, using genetic variants as instrumental variables to estimate causal effects in observational studies.^[8,9]^ The theoretical basis for MR comes from 2 fundamental principles of genetics: the distribution of genetic variants in a population is random, and these variants are unrelated to lifestyle and environmental factors. Therefore, if a genetic variant is associated with a potential risk factor that, in turn, affects disease development, that variant can serve as an instrumental variable to help us understand the causal relationship between the risk factor and the disease.^[10]^ In this way, MR creates a natural randomized controlled experiment, allowing researchers to assess the impact of an exposure factor (such as lifestyle, biomarkers, etc.) on health outcomes without actual intervention. With advancements in bioinformatics and statistical methods, MR studies can now incorporate multiple genetic variants, increasing the precision and power of the analyses. For example, researchers can estimate the causal impact of certain biomarkers on disease risk using one or multiple genetic variants associated with these biomarkers. This approach has been employed to assess relationships between factors such as lipid levels, blood pressure, body mass index (BMI), and health outcomes like cardiovascular diseases, diabetes, and others.^[11]^ MR is now widely used in academic research and is gradually becoming a powerful tool in drug development and public health decision-making. By revealing potential causal relationships, MR helps to prioritize intervention strategies and avoid wasting resources on ineffective or harmful treatments.^[12]^ In summary, Mendelian Randomization is a powerful epidemiological tool that uses genetic variants to reveal causal relationships between exposure factors and disease outcomes. With the growing richness of genetic data and continual advancement of analytical methods, the application of MR is promising and expected to play an even greater role in future medical research and practice.

Despite pulmonary fibrosis being a potentially fatal lung disease, its pathophysiological mechanisms are not fully understood, especially regarding the specific role of immune cells in disease progression. Early research has revealed the potential importance of immune regulation in pulmonary fibrosis, but traditional epidemiological methods struggle to differentiate whether immune cell changes are a cause or a result of the disease. Moreover, there is an urgent need to establish a causal relationship between immune factors and pulmonary fibrosis in the exploration of potential treatment targets. In this context, this paper employs the Mendelian Randomization approach, a method using genetic variants as tools in a natural experiment to assess the causal relationship between immune cells and pulmonary fibrosis. The unique advantage of the MR method is that it can provide evidence of causality in the relationship between immune cells and pulmonary fibrosis, unaffected by confounding factors. By utilizing genetic variants associated with the number or function of immune cells, we can evaluate the impact of these immune cell characteristics on the risk of pulmonary fibrosis while avoiding issues of reverse causality and confounders common in traditional observational studies. Furthermore, given that there has been no literature report using Mendelian Randomization to study this specific relationship to date, this study offers a novel perspective and could potentially pave new pathways for immunoregulatory treatment of pulmonary fibrosis. In conclusion, this study aims to fill the gap in current research, providing a clear causal picture of the role of immune cells in pulmonary fibrosis through innovative Mendelian Randomization analysis. This not only enhances our understanding of the pathophysiological mechanisms of pulmonary fibrosis but may also provide a scientific basis for the development of new therapeutic strategies.

## 2. Materials and methods

### 2.1. Data source

Our study utilized the public GWAS database Open GWAS for data collection.^[13]^ We selected the most comprehensive dataset for whole-genome association studies on peripheral blood immune phenotypes. This dataset includes 118 absolute cell count indicators, 389 median fluorescence intensity indicators reflecting cell surface antigen levels, 32 morphological parameters related to cell volume and internal complexity (such as forward and side scatter), and 192 relative cell count indicators. The summary statistics for these 731 immune traits are publicly available in the GWAS Catalog, with access numbers ranging from GCST0001391 to GCST0002121.^[14]^ The GWAS summary data on pulmonary fibrosis used in our study were obtained from the Open GWAS database. This dataset, identified by the code ebi-a-GCST90018908, covers 469,126 individuals of European descent, including 1566 cases of pulmonary fibrosis and 467,560 healthy controls. Given that all data are from openly accessible databases, our study did not require additional approval from independent ethical review. This method of data acquisition ensures the transparency and credibility of our research, as well as promoting the widespread dissemination and exchange of our findings within the scientific community.

### 2.2. Selection of instrumental variables

In the preliminary steps of our Mendelian Randomization (MR) analysis, we first selected single nucleotide polymorphisms (SNPs) associated with specific exposures that reached genome-wide statistical significance (*P* value less than 5 × 10^−8^) to serve as our instrumental variables (IVs). To ensure the independence of these SNPs, we further screened for linkage disequilibrium, retaining only those SNPs with low linkage disequilibrium (r^2^ < 0.001) and physically positioned more than 10,000 base pairs apart. We also calculated the F-statistic for each potential IV to measure its association with the exposure variable, discarding any IV with an F-statistic <10 to enhance the validity of our instrumental variables in the analysis. To ensure these IVs did not indirectly affect our study outcomes through pleiotropic pathways, we used the PhenoScanner database to cross-reference each IV to identify and exclude those possibly associated with pleiotropic traits. This rigorous selection process strengthens the accuracy and credibility of our MR analysis, ensuring the robustness of the conclusions drawn.

### 2.3. Statistical analysis

In this study, to establish the causal effects of exposure factors on outcomes, we employed a range of Mendelian Randomization (MR) analysis techniques. These included Inverse Variance Weighted (IVW) method, Weighted Median approach, MR-Egger method, Simple mode, and Weighted mode.^[15,16]^ Each technique relies on different statistical assumptions to offer multi-faceted causal inference. The IVW method assumes all SNPs are valid instruments unaffected by pleiotropy and uses the inverse variance of each result as weights for estimation. MR-Egger is employed to detect the impact of pleiotropy and provides adjusted estimates in the presence of pleiotropic effects. The Weighted Median approach, under the premise that at least half of the instrumental variables are valid, offers a more robust causal estimate. Meanwhile, Simple mode and Weighted mode estimate causal relationships by simulating simplified randomized tests and using a weighted IV approach, respectively.

We used Cochran’s Q statistic to assess heterogeneity in causal estimates from the IVW and MR-Egger methods, considering a *P* value below .05 as significant for heterogeneity. Additionally, leave-one-out analysis was conducted to detect pleiotropy of instrumental variables, where each SNP was removed one by one, repeating the MR analysis with the remaining SNPs to identify any outliers. All MR analyses were conducted in R software version 4.2.2 using the “TwoSampleMR” and “MR-PRESSO” packages. These comprehensive methods and validation measures enhance the stability and scientific value of our study conclusions, providing a thorough evaluation of the causal link between exposure factors and outcomes.

## 3. Results

### 3.1. Heterogeneity analysis

Our study performed a heterogeneity analysis on 1462 genetic loci to assess the consistency of the association between immune cells and pulmonary fibrosis. The results revealed statistically significant differences among these genetic loci, with a wide distribution of Q statistics ranging from 0.027 to 750.35 (Supplementary File 1, http://links.lww.com/MD/L205). This indicates varying degrees of heterogeneity in the association between different types of immune cells and pulmonary fibrosis, likely due to differences in biological mechanisms. For instance, macrophages, T cells, and B cells might participate in the inflammation and repair processes of lung tissue through distinct pathways, thus playing different roles in disease progression. Additionally, we observed that while the median Q value for most loci was 21.20, indicating a general level of heterogeneity, some loci had Q values significantly higher than the 75th percentile (27.98), suggesting these loci could be key genetic variations in the development of pulmonary fibrosis that warrant further study. Finally, we excluded immune cells with significant heterogeneity (*P* < .05) for further analysis.

### 3.2. Pleiotropy analysis

The Egger regression method was employed to test for pleiotropy. Analysis of 731 SNP loci indicated an average intercept close to zero, suggesting that the instrumental variables chosen were statistically robust (Supplementary File 2, http://links.lww.com/MD/L206). Nevertheless, due to a standard deviation of the regression intercept of 0.0226, individual SNP intercepts were observed to deviate significantly from zero in certain cases, which may indicate the presence of pleiotropy under specific conditions. Immune cells with significant pleiotropy (*P* < .05) were excluded for subsequent analyses.

### 3.3. Mendelian randomization analysis

**Figure 1.**
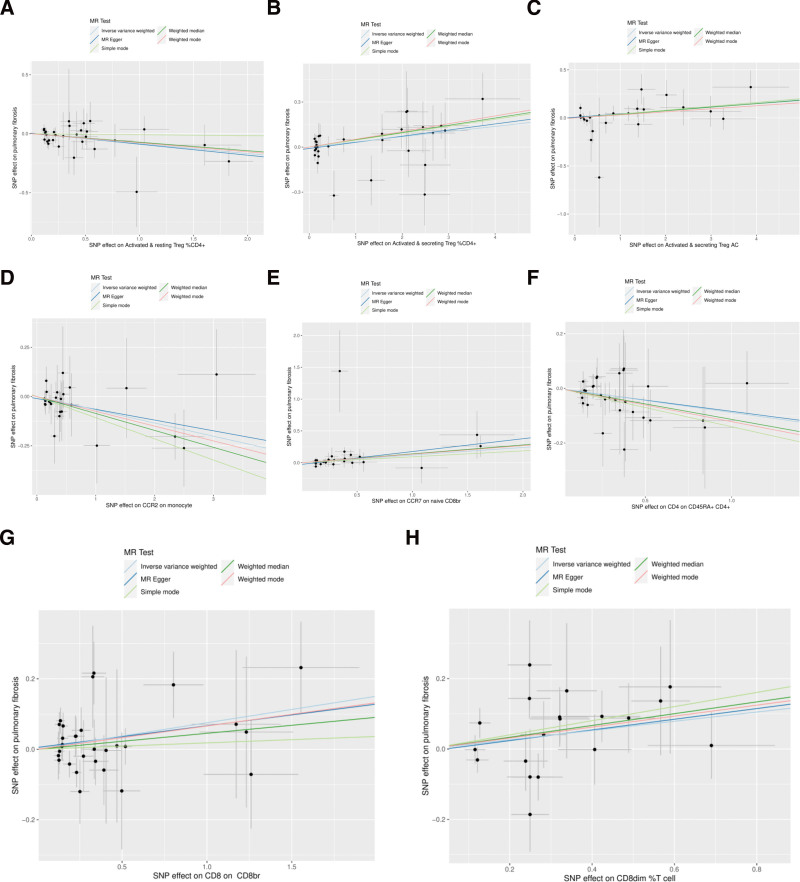
Scatterplot of Mendelian randomization methods for immune cells. Scatterplot of Activated & resting Treg _CD4 + (A), Activated & secreting Treg _CD4 + (B), Activated & secreting Treg AC (C), CCR2 on monocyte (D), CCR7 on naive CD8br (E), CD4 on CD45RA + CD4 + (F), CD8 on CD8br (G), CD8dim _T cell (H).

**Figure 2.**
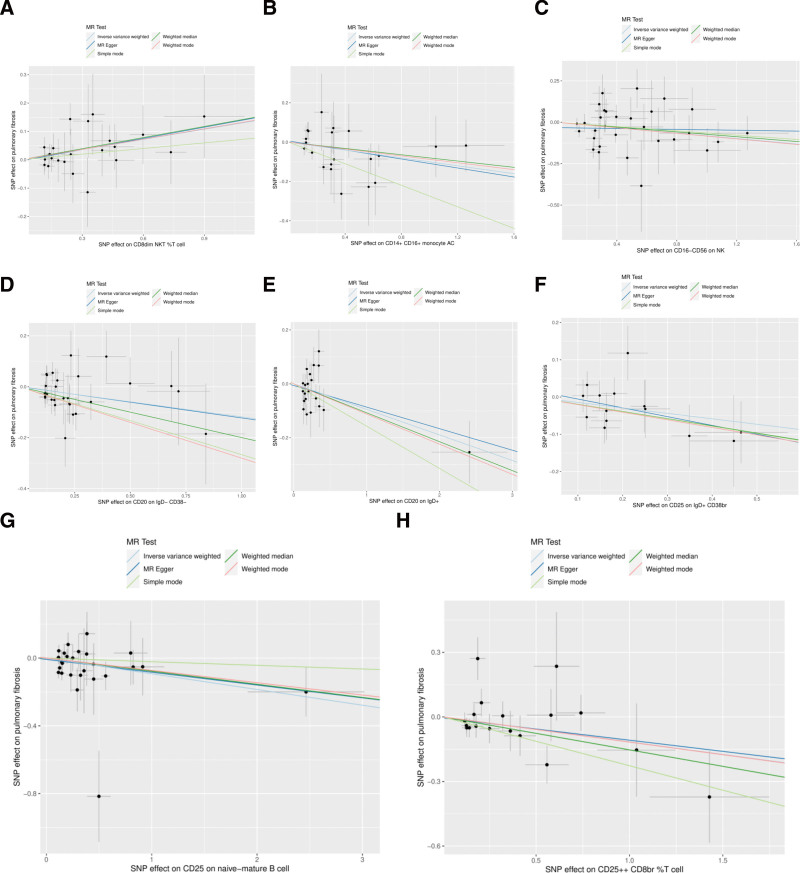
Scatterplot of Mendelian randomization methods for immune cells. Scatterplot of CD8dim NKT _T cell (A), CD14 + CD16 + monocyte AC (B), CD16-CD56 on NK (C), CD20 on IgD- CD38- (D), CD20 on IgD + (E), CD25 on IgD + CD38br (F), CD25 on naive-mature B cell (G), CD25++ CD8br _T cell (H).

**Figure 3.**
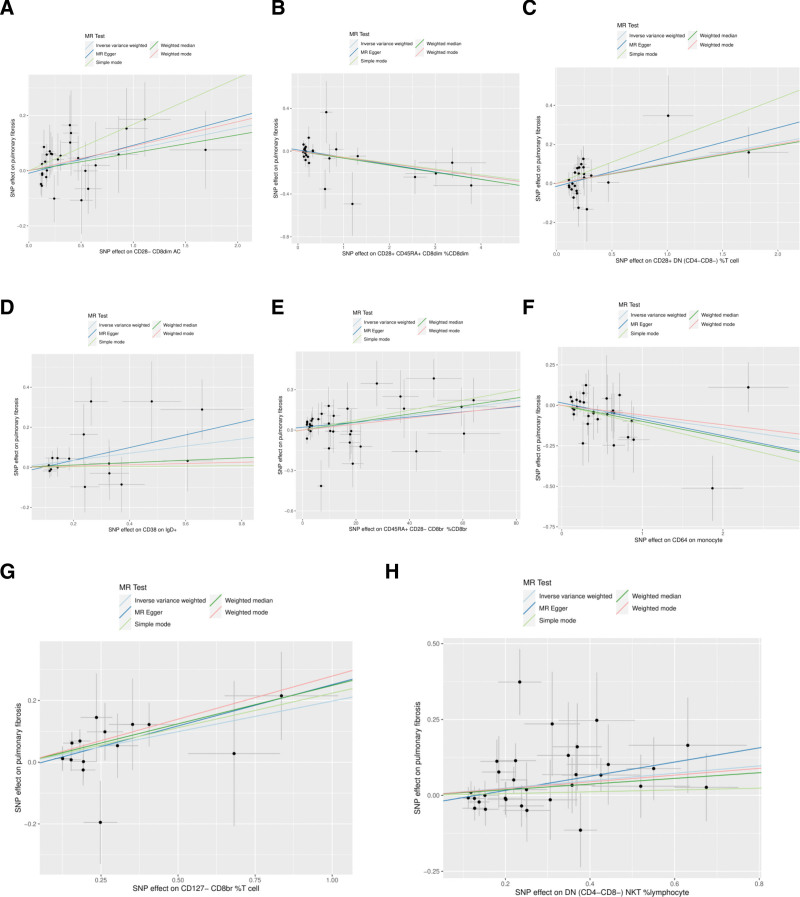
Scatterplot of Mendelian randomization methods for immune cells. Scatterplot of CD28- CD8dim AC (A), CD28 + CD45RA + CD8dim _CD8dim (B), CD28 + DN (CD4-CD8-) _T cell (C), CD38 on IgD + (D), CD45RA + CD28- CD8br CD8br (E), CD64 on monocyte (F), CD127- CD8br _T cell (G), DN (CD4-CD8-) NKT _lymphocyte (H).

**Figure 4.**
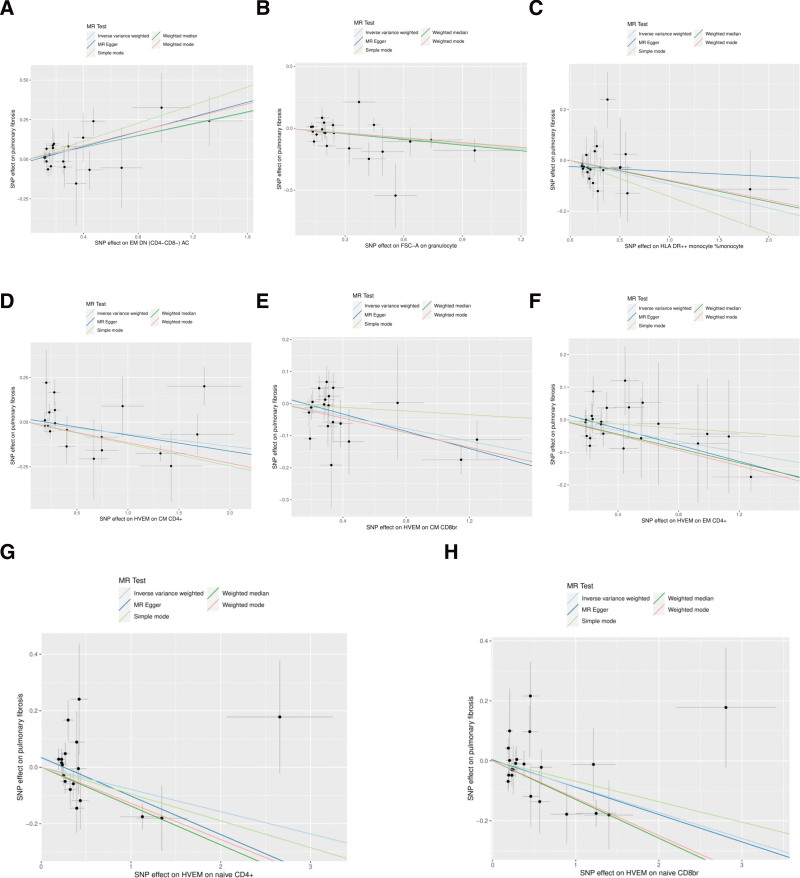
Scatterplot of Mendelian randomization methods for immune cells. Scatterplot of EM DN (CD4-CD8-) AC (A), FSC-A on granulocyte (B), HLA DR++ monocyte _monocyte (C), HVEM on CM CD4 + (D), HVEM on CM CD8br (E), HVEM on EM CD4 + (F), HVEM on naive CD4 + (G), HVEM on naive CD8br (H).

**Figure 5.**
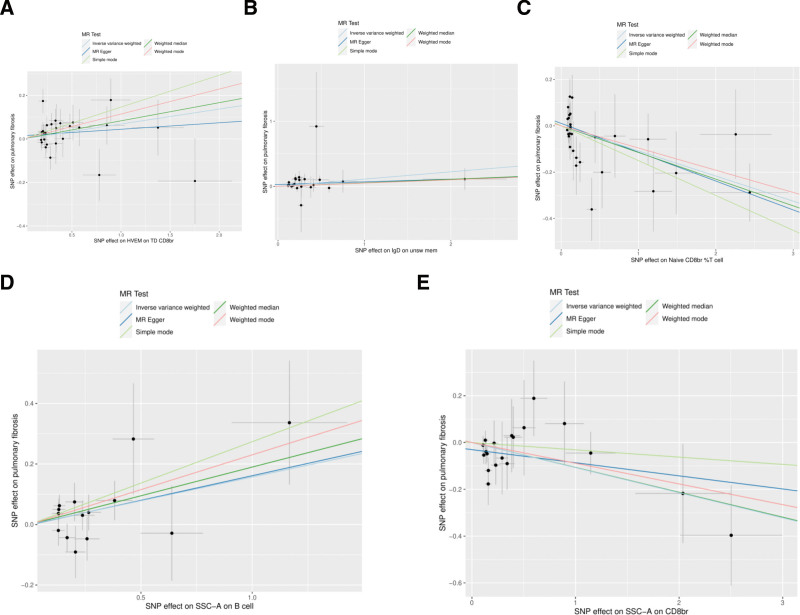
Scatterplot of Mendelian randomization methods for immune cells. Scatterplot of HVEM on TD CD8br (A), IgD on unsw mem (B), Naive CD8br _T cell (C), SSC-A on B cell (D), SSC-A on CD8br (E).

**Figure 6.**
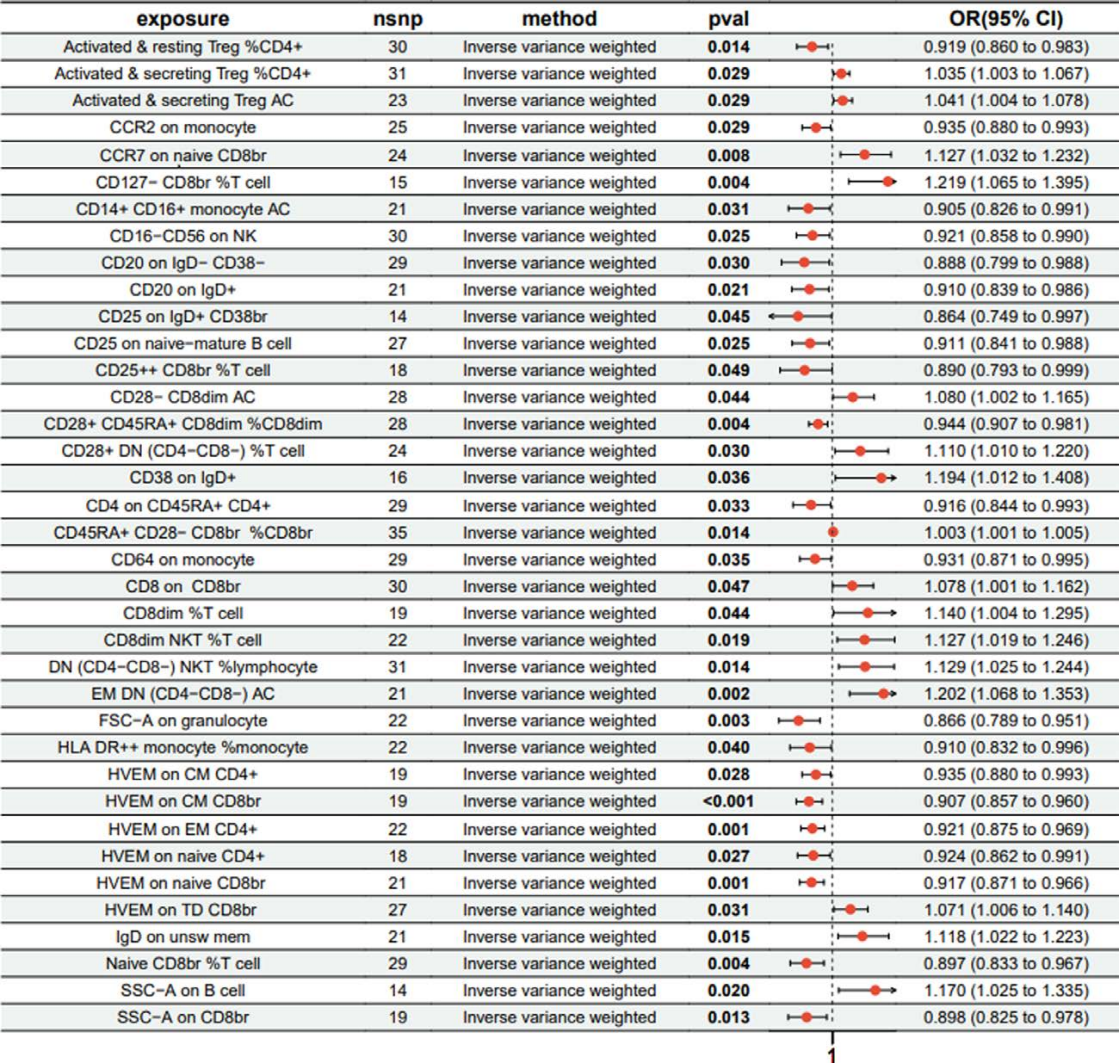
Forest diagram of 37 immune cells.

In the MR analysis, a comprehensive analysis of 18,193 single nucleotide polymorphisms (SNPs) was performed to evaluate the association between genetic variations in immune cells and the phenotype of pulmonary fibrosis (Supplementary File 3, http://links.lww.com/MD/L207). The average effect size (beta value) of these SNPs was 0.1282, indicating a generally positive correlation between most SNPs and the phenotype of immune cells. However, the average *P* value for the outcome effect was .492956, which suggests that these loci are not statistically significantly associated with pulmonary fibrosis directly. This may reflect the multifactorial nature of pulmonary fibrosis, with its genetic underpinnings involving complex interactions between multiple genes and environmental factors. According to the final results, a total of 37 types of T cells, B cells, natural killer cells, and monocytes showed a significant causal relationship with pulmonary fibrosis. Specifically, certain immune cells like activated & resting Treg %CD4+, CCR2 on monocytes, and CD4 on CD45RA + CD4 + were identified as protective factors against pulmonary fibrosis (beta < 0). Conversely, activated & secreting Treg _CD4+, CCR7 on naive CD8bright, and CD8 on CD8bright cells were among those identified as risk factors (beta > 0) (Supplementary File 4, http://links.lww.com/MD/L208). Additionally, scatter plots were constructed to visualize the relationship between the 37 types of immune cells and pulmonary fibrosis (Figs. 1–5). Leave-one-out analyses were performed for each of the 37 immune cell types, followed by forest plots construction, and tests for heterogeneity and pleiotropy, with negative results for both. Lastly, a forest plot was constructed based on the IVW method for the relationship between the 37 immune cells and pulmonary fibrosis (Fig. 6). Taken together, these results substantiate a significant causal relationship between the 37 types of immune cells and pulmonary fibrosis.

## 4. Discussion

Pulmonary fibrosis is a chronic, progressive lung disease characterized by excessive scarring of lung tissue, which results in the replacement of normal pulmonary architecture with fibrotic tissue, thereby affecting lung function and causing breathing difficulties. Although researchers have identified a variety of risk factors associated with pulmonary fibrosis, including genetic predispositions, environmental exposures, and autoimmune processes, the specific pathophysiological mechanisms remain unclear.^[17]^ In this context, exploring the role of the immune system in the pathogenesis of pulmonary fibrosis is crucial for understanding the nature of the disease and developing new therapeutic approaches. Immune cells, as vital components of the body’s defense system, have been proven to play a key role in the progression of many diseases.^[18]^ Within the pathology of pulmonary fibrosis, regulatory T cells (Tregs), natural killer (NK) cells, and different subtypes of monocytes (such as M1 and M2 macrophages) are considered to have potential significant roles.^[19]^ However, the evidence for a causal relationship between these immune cells and pulmonary fibrosis is still insufficient. Traditional observational studies are limited by confounding factors and reverse causation, which do not clarify the direct link between immune cell characteristics and pulmonary fibrosis. MR is a method that uses genotypes as instrumental variables to assess the causal relationship between exposure factors and disease outcomes. Since genotypes are determined at conception and are generally not affected by lifestyle or environmental factors, the MR method can theoretically overcome some of the limitations of traditional observational studies, such as confounding factors and reverse causation issues.^[20]^ Therefore, this study employed the MR approach to systematically investigate the causal relationship between immune cell characteristics and the risk of pulmonary fibrosis, aiming to provide new insights for the prevention and treatment of pulmonary fibrosis.

This study, using the Mendelian randomization approach, systematically investigates for the first time the causal relationship between immune cells and pulmonary fibrosis. Our analysis, based on large-scale genetic and phenotypic data, reveals a direct connection between specific immune cell characteristics and the development of pulmonary fibrosis. We have found that various types of immune cells, including regulatory T cells, natural killer cells, and monocytes, have a significant causal association with the risk of pulmonary fibrosis. These findings not only provide new insights into the pathophysiological mechanisms of pulmonary fibrosis but may also lay the theoretical groundwork for developing targeted therapeutic strategies. Regulatory T cells (Tregs) play a crucial role in the development of pulmonary fibrosis, not only because they maintain an appropriate balance of immune response but also because they can suppress the overactivation that may lead to tissue damage. Tregs inhibit the activation and proliferation of inflammatory cells by releasing anti-inflammatory cytokines, such as transforming growth factor-beta (TGF-β) and interleukin-10 (IL-10), thereby preventing a spiraling inflammatory response.^[21]^ Furthermore, Tregs can also directly interact with effector T cells and inhibit their function through cell-contact-dependent mechanisms.^[22]^ In the case of pulmonary fibrosis, an increase in the ratio of activated & resting Treg %CD4 + may represent a protective mechanism to prevent the initiation and progression of fibrosis, a point supported by our research. This protective discovery reminds us that enhancing the function of Tregs could be an effective way to prevent or reverse pulmonary fibrosis in future therapeutic strategies. The dual function of natural killer (NK) cells is equally critical in pulmonary fibrosis. They can directly kill virus-infected cells and tumor cells and regulate immune responses by releasing cytokines such as interferon-gamma (IFN-γ) and tumor necrosis factor-alpha (TNF-α).^[23]^ In the environment of pulmonary fibrosis, this regulation may lead NK cells to shift from promoting inflammation to aiding tissue repair, possibly by affecting the phenotype and function of other immune cells. Our data indicates that the phenotype of CD16-CD56 positive NK cells is associated with a reduced risk of pulmonary fibrosis, suggesting that NK cells play a positive role in antifibrosis by mitigating tissue damage and promoting tissue repair. This finding provides a new perspective on the potential role of NK cells in the prevention and treatment of pulmonary fibrosis. Macrophages exhibit a dual nature in the development of pulmonary fibrosis. They can differentiate into pro-inflammatory M1 or reparative M2 types based on signals from their environment. M1 macrophages are usually associated with inflammation and tissue destruction, whereas M2 macrophages are involved in tissue repair and fibrosis.^[24]^ Our study reveals that CCR2 positive monocytes are related to a reduced risk of pulmonary fibrosis, possibly indicating a shift towards an M2 phenotype, thereby playing a role in inhibiting the pathological process of pulmonary fibrosis. This shift in immune cell phenotype emphasizes that modulating macrophage activity could be an important target in treating pulmonary fibrosis. However, there are also subgroups of immune cells that are associated with an increased risk of pulmonary fibrosis. This may suggest that certain immune cells may have a negative role in promoting inflammation and fibrotic deposition, such as certain CD8 + T cell subgroups. Their activation could lead to the release of inflammatory factors, exacerbating lung tissue damage and fibrosis. Thus, our findings highlight a complex network of immune cells containing different roles that both inhibit and promote fibrosis. Notably, we also found some immune cell phenotypes associated with an increased risk of pulmonary fibrosis, suggesting the activation of pro-inflammatory and pro-fibrotic immune cells in the development of this disease. For instance, certain subgroups of CD8 + T cells have been shown to be a risk factor for pulmonary fibrosis. These findings hint that the role of immune cells in pulmonary fibrosis is complex and multifaceted, not simply a single effect.

However, our study does have certain limitations. Firstly, since our analysis depends on genetic association data from public databases, the quality and accuracy of these data are critical to our findings. Secondly, Mendelian randomization assumes that the instrumental variables influence outcomes solely by affecting the specified exposure and not through confounding factors or pleiotropic pathways. While we have attempted to mitigate these factors through various means, we cannot completely eliminate all potential interferences. Additionally, our study subjects were mainly from a European background, which may limit the universality of our results. Future studies will need to further verify these findings in populations of different ethnicities and geographical areas. Clinical trials should also contemplate the potential application of immunomodulatory treatments in the prevention or treatment of pulmonary fibrosis. Moreover, with the increasing richness of individual genomic data, future MR studies will be able to more precisely investigate the relationships between genetic variations and immune cell functions, thus offering support for personalized medicine.

## 5. Conclusion

This study, utilizing the Mendelian randomization approach, has systematically explored the causal relationship between immune cell characteristics and pulmonary fibrosis for the first time. Our results emphasize the potential protective roles of regulatory T cells (Tregs), natural killer (NK) cells, and M2 macrophages in the pathophysiology of pulmonary fibrosis. Specifically, increased levels of Tregs, CD16-CD56 positive NK cell phenotypes, and CCR2 positive monocytes are associated with a decreased risk of pulmonary fibrosis, potentially exerting protective effects by inhibiting inflammatory responses and promoting tissue repair. Additionally, certain subgroups of CD14 + CD16 + monocytes have also demonstrated a protective effect, which further confirms the complex role of immune cells in modulating pulmonary fibrosis. Conversely, subgroups of CD8 + T cells have been associated with an increased risk of pulmonary fibrosis, potentially reflecting their role in promoting inflammation and fibrotic deposition. Overall, this study not only provides new scientific evidence for the prevention and treatment of pulmonary fibrosis but also highlights the importance of further research to deepen our understanding of the roles immune cells play in this disease, ultimately aiming to improve the clinical treatment options for patients.

## Author contributions

**Conceptualization:** Zhiyu Tian, Li Shi.

**Data curation:** Zhiyu Tian.

**Formal analysis:** Zhiyu Tian, Li Shi.

**Investigation:** Zhiyu Tian.

**Methodology:** Zhiyu Tian, Shaodan Hu.

**Project administration:** Zhiyu Tian, Shaodan Hu.

**Resources:** Zhanliang Jiang, Shaodan Hu.

**Validation:** Zhanliang Jiang, Li Shi.

**Visualization:** Zhanliang Jiang, Li Shi.

**Writing – original draft:** Zhiyu Tian, Zhanliang Jiang, Li Shi, Shaodan Hu.

**Writing – review & editing:** Zhanliang Jiang, Li Shi.

## Supplementary Material








